# Urban green spaces and healthy aging: the role of physical activity

**DOI:** 10.3389/fpubh.2026.1785881

**Published:** 2026-03-23

**Authors:** Xu Wang, Chengyue Li, Xuguang Wang

**Affiliations:** Research Center of Sports Humanities and Social Sciences (National Sports and Fitness Research Think Tank), Tianjin University of Sport, Tianjin, China

**Keywords:** environmental behavior science, healthy aging, regional disparity, physical activity, urban green spaces

## Abstract

**Background:**

Against the backdrop of ongoing urban renewal and the Healthy China initiative, examining the relationship between urban green spaces, physical activity, and healthy aging among residents holds significant importance for advancing urban health transformation and refining public health governance systems.

**Methods:**

Utilising provincial panel data from 28 Chinese provinces spanning 2010–2023, this study employed a two-way fixed effects model and mediation analysis to examine the statistical association between urban green spaces, physical activity, and healthy aging, along with their behavioural transmission pathways, while conducting robustness tests.

**Results:**

(1) Urban green spaces levels were significantly positively correlated with healthy aging among residents (β = 0.122, *p* < 0.01). (2) Urban green spaces was significantly positively correlated with residents’ physical activity levels (β = 0.029, 95% CI [0.006, 0.053], *p* < 0.05). (3) Physical activity exhibited a partial mediating pathway between urban green spaces and residents’ healthy aging. Physical activity was significantly positively correlated with residents’ healthy aging (β = 0.695, 95% CI [0.303, 1.087], *p* < 0.01); the total effect of urban green spaces on healthy aging was significantly positively correlated (β = 0.122, 95% CI [0.048, 0.196], *p* < 0.01), with a significant positive direct effect (β = 0.102, 95% CI [0.027, 0.177], *p* < 0.01) and a statistically significant indirect effect (β = 0.020, 95% CI [0.002, 0.039], *p* < 0.05). (4) Significant regional differences emerged across income levels. In high-income areas, urban green spaces exhibited a significant positive correlation with healthy aging (β = 0.178, *p* < 0.01); in low-income areas, this relationship failed to reach statistical significance (β = 0.026, *p* > 0.10). Concurrently, physical activity exhibited a significant positive correlation with healthy aging in both income categories, though its effect was stronger in low-income areas (low-income areas: β = 0.750, *p* < 0.05; high-income areas: β = 0.678, *p* < 0.05).

**Conclusion:**

Urban green spaces not only directly promote healthy aging but also exert an indirect effect by enhancing physical activity levels. This study elucidates the associative mechanisms linking urban green spaces, physical activity, and healthy aging, providing empirical evidence for optimising urban green space development, promoting resident physical activity engagement, and refining policies related to healthy aging.

## 1 Introduction

With China’s rapid economic growth and ongoing urbanization, urban lifestyles have undergone significant transformations, presenting new health challenges for the older adults in housing, mobility, and social engagement. Presently, China has entered a moderately aged society and is accelerating towards deep ageing, where issues such as multiple coexisting conditions, rising disability risks, insufficient physical activity, and heightened psychological pressures among older adults are increasingly prominent (1). Against this backdrop, how to leverage sustainable and accessible environmental factors to promote healthy aging has become a critical issue in contemporary population governance. Green spaces constitute a vital component of ecological civilization development and serve as essential public resources for enhancing urban liveability and supporting residents’ physical and mental wellbeing. Since the 18th National Congress of the Communist Party of China, China’s urban green space development has advanced steadily, with significant increases in both the greening coverage rate of built-up areas and the per capita park green space area. The data shows that by 2021,the green coverage rate in China’s urban built-up areas had increased from 39.22% in 2012 to 42.06%, while the per capita park green space area rose from 11.80m^2^ to 14.78m^2^. Urban green spaces are playing an increasingly vital role in improving air quality, optimizing microclimates, and providing nature-based experiences (2). As the ecological, recreational and health benefits of green spaces become increasingly evident, their potential role in meeting the health needs of older adults, enhancing daily activity environments and supporting active lifestyles is gaining attention. They have thus emerged as an indispensable environmental foundation for promoting healthy aging.

In recent years, academic discourse has explored the health effects of urban green spaces from multiple perspectives, primarily focusing on three areas. Firstly, studies examining natural exposure have emphasized the direct impact of green spaces on physical and mental wellbeing (3), yet insufficient attention has been paid to their long-term, structural effects. Secondly, analyses from the perspective of spatial accessibility have investigated the spatial matching relationship between green space provision and the distribution of ageing populations (4), though these have predominantly centered on urban case studies, lacking longitudinal quantitative verification of green space health effects. Thirdly, studies have examined the mechanisms through which green spaces influence active ageing via built environment improvements (5), yet research systematically revealing these mechanisms from a physical activity participation perspective remains scarce. Environmental behavioral science indicates that health behaviors, particularly physical activity engagement, constitute key pathways through which urban green spaces enhance wellbeing. Physical activity participation improves physical fitness, bolsters psychological resilience, and strengthens social connections (6, 7), aligning closely with core elements of healthy aging. At the policy level, documents such as the Healthy China 2030 Plan Outline and the National Fitness Programme (2021–2025) explicitly advocate for the deep integration of national fitness initiatives with ecological civilization development. They propose leveraging ecological resources like parks, greenways, and waterside spaces to create accessible, user-friendly, and multifunctional fitness environments, thereby encouraging regular physical activity among middle-aged and older adults. However, systematic empirical analysis remains lacking regarding the associative structure and behavioral transmission pathways linking urban green space development with physical activity in healthy aging. To address this, this study employs panel data from 28 Chinese provinces spanning 2010–2023 to construct a two-way fixed effects model and a panel mediation model. This enables a systematic analysis of the overall effects, behavioral pathways, mediating mechanisms, and regional variations of urban green spaces in promoting healthy aging. Specifically, this paper addresses the following four research questions: RQ1: Does the level of urban green space provision significantly predict healthy aging at the provincial level? RQ2: Is there a significant correlation between urban green space provision and physical activity among older adults? RQ3: Does physical activity mediate the relationship between urban green space and healthy aging? RQ4: Does the relationship between urban green space and healthy aging exhibit significant regional variation across areas with differing levels of economic development? Building upon this, the paper formulates corresponding research hypotheses and tests them through empirical analysis, aiming to provide insights for healthy city development, green space planning, and the optimization of national fitness policies.

## 2 Review

### 2.1 Environmental behavior theory

Environmental behavior theory emerged in the 1960s as an interdisciplinary theoretical framework synthesizing environmental science, architecture, social psychology, and urban planning. Its core proposition lies in elucidating the dynamic interaction mechanism between ‘environment, behavior, and outcome’ (8). This theory posits that individual behavior does not occur in isolation but constitutes an adaptive response shaped by specific physical and social environments. Concurrently, the accumulation of human behavior reciprocally molds the functional attributes and usage patterns of spatial environments, forming a continuous feedback loop. Through progressive research, this theoretical framework has evolved into three fundamental perspectives: environmental determinism, interactionism, and mutual permeation.

Early environmental determinism emphasized the direct shaping influence of the physical environment on human behavior, positing that objective environmental factors such as light exposure, climate, vegetation coverage, and spatial structure could directly impact behavioral responses and activity tendencies at both physiological and psychological levels. From this perspective, urban green spaces are regarded as vital natural elements for promoting residents’ health. High-quality green spaces can directly enhance the physical and mental wellbeing of older adults by improving air quality, reducing noise disturbance, and increasing visual comfort, thereby providing foundational environmental support for healthy aging (9). As research deepened, the interactionist theory posited that behavior arises from the combined influence of individual characteristics and environmental conditions. It contends that human behavior is not solely constrained by external environments but is also shaped by factors such as individual motivation, health status, values, and social support. Regarding urban green spaces, their spatial openness, accessibility, and safety provide essential conditions for residents’ participation in physical activities. However, whether residents actually engage in such activities is further influenced by multiple factors including physical capabilities, exercise habits, willingness to participate, and social relationships (10). The theory of mutual permeation emphasizes that the relationship between people, environment, and behavior is neither linear nor static, but rather an evolving holistic system over time. The environment not only influences behavior; behavior, in turn, endows the environment with new meanings and reshapes the social functions and emotional attributes of space through repeated use. For instance, urban green spaces, through long-term hosting of residents’ walking, exercise, and social activities, gradually cultivate specific spatial cultures and collective memories, further enhancing the environment’s appeal and behavioral incentive effects. The sustained occurrence of physical participation not only improves individual physical health and psychological well-being but also fosters social connections and neighborhood interactions, rendering green spaces vital social arenas for promoting health, vitality, and engagement among older adults. In summary, environmental behavioral science systematically reveals the multi-layered connections between environmental characteristics, behavioral processes, and health outcomes, providing a fundamental theoretical framework for explaining how urban green spaces promote healthy aging through increased physical activity participation. These spaces not only directly influence physical and mental wellbeing through their ecological attributes but also indirectly impact older adults’ health levels via pathways such as behavioral incentives, emotional restoration, and social interaction.

### 2.2 Research hypotheses

#### 2.2.1 Impact of urban green spaces on healthy aging

Healthy aging is a multidimensional outcome state, signifying that older adults can, through proactive resource mobilization and situational adaptation during the ageing process, minimize health risks, maintain high levels of physical and mental functioning, and achieve sustained social participation (11). According to the World Health Organisation (WHO) framework for healthy aging, its core lies in the maintenance of functional ability – that is, an individual’s capacity to achieve their valued lifestyle within their supportive environment, determined by the interaction between their inherent capabilities and their surroundings (12, 13). Concurrently, the life course perspective posits that healthy aging is not an isolated outcome of the older adults stage, but rather the manifestation of long-term cumulative influences from early developmental conditions, social structural environments, behavioral pathways, and environmental exposures. Its key lies in understanding how ‘risk accumulation’ and ‘critical period exposure’ shape functional trajectories in later life. At the measurement level, existing research predominantly employs multidimensional frameworks to define healthy aging, aligning with the three pillars of ‘health, participation, and security’ emphasized by the WHO framework (14). This primarily encompasses three core dimensions: physical health, mental health, and social participation (15, 16). Physical health reflects an individual’s capacity for maintaining bodily functions and managing chronic conditions; mental health indicates emotional regulation and psychological resilience; while social participation measures older adults’ interactions with external society and functional continuity. These three dimensions interact synergistically, collectively forming the foundation of overall well-being during the later stages of life.

Urban green spaces typically refer to open areas within the built environment that feature natural vegetation cover, such as parks, community green spaces, and urban forests. As a vital component of the urban ecosystem, these green spaces influence individual health through mechanisms including microclimate regulation, air purification, and the promotion of physical activity and psychological recovery. At the measurement level, research commonly distinguishes between subjective and objective indicators. Subjective green space metrics are often assessed through questionnaires evaluating residents’ perceptions of accessibility, adequacy, and maintenance conditions (17). Objective measurements primarily rely on remote sensing and GIS data, including indicators such as the Normalised Difference Vegetation Index (NDVI), Enhanced Vegetation Index (EVI), and Land Use/Land Cover (LULC) (18). In recent years, research has begun integrating GPS trajectory data to evaluate urban green spaces, typically measured across three dimensions: availability, accessibility, and visibility. Methods employed include buffer zone analysis, network distance calculation, and green visibility rates based on street-level imagery.

Contemporary environmental health theory posits that the quality of an individual’s natural surroundings not only influences their physiological functions and psychological state, but also continuously shapes their health behavior patterns throughout the life course, thereby exerting a profound impact on health and social functioning in later life. Life course research emphasizes the dynamic interplay between structural conditions and individual agency, highlighting that personal health trajectories are shaped progressively through the long-term interplay of multiple factors including institutional environments, familial roles, and social opportunity structures. For instance, studies employing intersectional life course perspectives reveal that structural factors such as migration trajectories and institutional support influence social participation and health accumulation processes at different life stages (19), demonstrating that healthy aging is not solely determined by physiological and behavioral factors but is profoundly constrained by environmental and institutional contexts. For older adults, urban green spaces not only provide ecological services such as air purification, cooling, and noise buffering, but also offer low-cost, accessible, and sustainable venues for outdoor activities and social interaction, constituting vital external conditions for achieving healthy aging (20). Existing research has explored the relationship between urban green spaces and residents’ physical and mental health, as well as social well-being, through perspectives such as green space accessibility, availability, and type. This has preliminarily validated the comprehensive health benefits of urban green spaces for older adults’ physical condition, emotional state, and social functioning. In terms of research design, early studies predominantly employed cross-sectional analyses, whereas recent years have seen a gradual shift towards longitudinal and quasi-experimental frameworks to enhance causal identification. For instance, fixed-effects models based on multi-year panel data have been utilized to analyze the dynamic relationship between green space changes and mental health or chronic disease risk (21); Some quasi-experimental studies have compared the effects of indoor versus outdoor green space training on competition anxiety among university athletes, finding that outdoor training significantly reduced anxiety levels while generating positive psychological effects through enhanced social cohesion and perceived green space quality (22). Despite the growing number of longitudinal and quasi-experimental studies, urban green space health research continues to face challenges from endogeneity and confounding factors. On the one hand, residential self-selection bias may lead individuals of higher socioeconomic status to reside in greener neighborhoods, thereby overestimating green space effects. On the other hand, factors such as income, education, air pollution, and community safety influence both green space distribution and health outcomes directly (23). Against this backdrop, while existing empirical research continues to expand, further refinement of identification strategies remains essential.

From a specific health perspective, existing evidence indicates that urban green spaces exert positive effects across multiple dimensions of healthy aging. Firstly, urban green spaces enhance the physical and mental wellbeing of older adults. Increasing green space area, vegetation coverage, and public funding for greening all improve residents’ physical and mental health alongside life satisfaction. Vulnerable groups derive greater benefits from these health effects, with mechanisms including improved environmental quality, expanded activity spaces, and enhanced social interaction (24). Secondly, urban green spaces possess unique advantages as semi-open social spaces in fostering older adults social participation. Samsudin R (2022) found that residents’ subjective perceptions of accessibility and environmental quality of nearby community green spaces promote social interaction behaviors and support the formation of social networks (25). Thirdly, from the perspective of restorative environments, natural settings receive more direct experimental support for their immediate improvement of mood. For instance, Feng Jianxi (26) demonstrated through electroencephalography that urban green spaces significantly increase alpha wave activity and reduce beta wave levels compared to noisy, busy urban areas, inducing a state of relaxation. This indicates green spaces possess direct mood-restorative effects, holding significant implications for the mental wellbeing of older adults.

Overall, existing research indicates that urban green spaces exert positive effects on healthy aging across multiple dimensions, including improving psychological well-being, enhancing physical activity levels, promoting social engagement, and increasing life satisfaction among older adults. Grounded in environmental psychology theory, urban green spaces not only yield short-term improvements in physical and mental health for older adults but also provide a stable health support system through long-term environmental exposure, thereby establishing a crucial environmental foundation for healthy aging. Accordingly, the following hypothesis is proposed:

*H1*: There exists a significant positive correlation between urban green space levels and healthy aging outcomes among older adults.

#### 2.2.2 The mediating role of physical participation in the impact of urban green spaces on healthy aging

Urban green spaces constitute not only vital components of the urban ecosystem but also core public spaces for residents to engage in outdoor physical exercise. The scale, layout quality, and accessibility of green spaces directly determine whether older adults can conveniently, safely, and consistently engage in physical activity. High-quality green environments improve air quality and microclimate, reducing physiological strain and psychological stress during exercise, thereby enhancing the appeal and participation frequency of physical activities.

Existing research indicates that residents’ demand for urban green spaces is expanding beyond mere recreational functions towards diverse purposes such as physical fitness and social interaction. From a spatial accessibility perspective, a significant negative correlation exists between green space proximity and physical activity: the closer a residence is to green areas, the higher the frequency of residents engaging in physical pursuits like walking or jogging (27). At the community level, enhancing the accessibility of park green spaces and strategically integrating fitness trails with sports facilities form a crucial foundation for creating health-friendly environments. This approach promotes residents’ daily physical activity and strengthens community health resilience (28). Historically, within the context of urban development and planning, sporting demands have also served as a significant driving force for green space renewal and morphological transformation. For instance, modern Nanjing incorporated extensive sports facilities, fitness trails, and children’s activity zones into its park system planning, thereby reinforcing the multifaceted role of green spaces in physical fitness, public recreation, and social education (29). Concurrently, demand-side research has corroborated this trend. Research indicates that over half of Beijing residents regard ‘insufficient sports facilities’ as a key constraint on green space utilization, with widespread expectations that adding facilities such as running tracks and fitness equipment would better meet their physical exercise needs (30).

**Figure 1:**
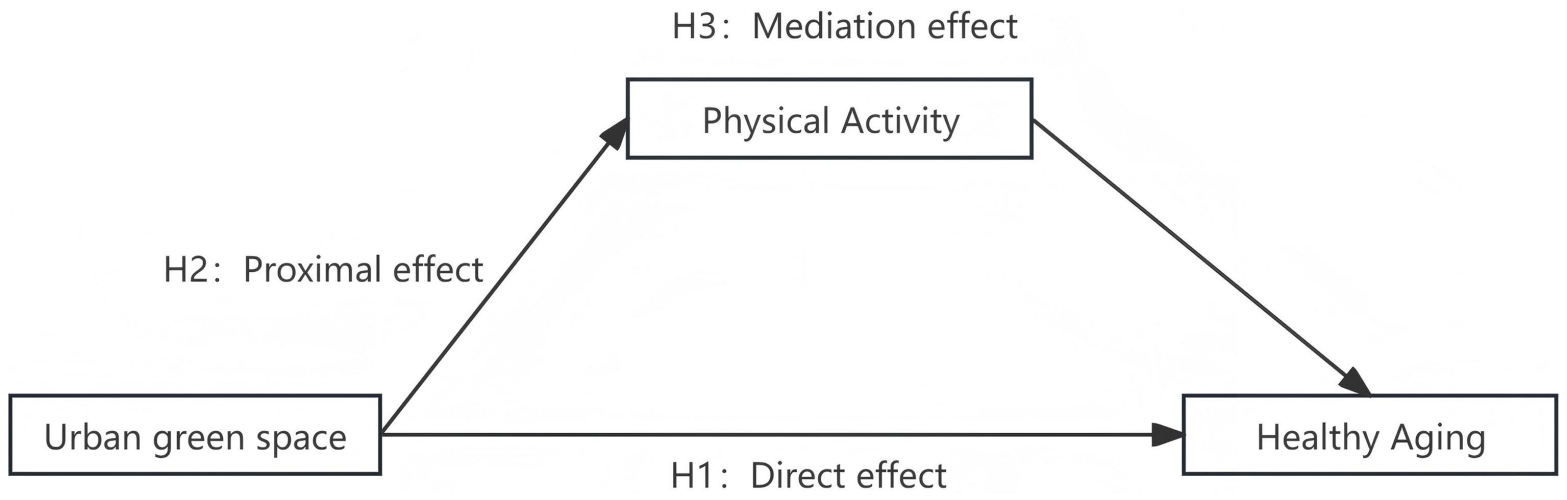
Theoretical framework.

Participation in physical activity, as a regular form of bodily engagement, serves as a vital means of promoting physical and mental wellbeing alongside functional maintenance. Environmental behavior theory indicates that individuals’ physical activity patterns are profoundly influenced by their physical surroundings. High-quality natural environments can significantly enhance residents’ participation rates and persistence in exercise by improving activity conditions, reducing the costs of physical exertion, and elevating the overall experience of movement. Concurrently, empirical research demonstrates that spatial characteristics of green spaces—such as scale, cleanliness, maintenance levels, and aesthetic appeal—not only influence physical activity frequency but also indirectly improve mental health by reducing residents’ stress levels (31–33). Furthermore, Yang Binghui (34) notes that urban green spaces alleviate the physiological and psychological burdens of smog by improving microclimates and providing safe, comfortable outdoor environments. By reducing perceived environmental risks, they also enhance willingness to engage in outdoor activities, thereby indirectly improving residents’ physical and mental wellbeing. For the older adults, green spaces offer suitable walking areas and venues for interaction, though individual physical condition, psychological security, and social needs also influence behavioral decisions. The superior air quality, comfortable microclimate, and visually restorative environments provided by green spaces help reduce the physiological and psychological burdens of exercise, enhancing both the enjoyment and persistence of physical participation among older adults. Simultaneously, green spaces fulfil rich social interaction functions. Group activities such as walking, tai chi, power walking, and square dancing often naturally form within these spaces. The accompanying neighborly exchanges, emotional support, and social motivation help enhance older adults’ social engagement and psychological wellbeing, indirectly promoting healthy aging. Based on these mechanisms, increasing urban green space may not only elevate older adults’ participation in physical activities but also indirectly influence residents’ healthy aging through this behavioral pathway (see Figure 1). Accordingly, the following hypotheses are proposed:

*H2*: The level of urban green space is significantly positively correlated with older adults’ participation in physical activities.

*H3*: Participation in physical activities mediates the relationship between urban green space and healthy aging among older adults.

#### 2.2.3 Regional heterogeneity hypothesis

Significant disparities exist in economic development levels across China’s provinces, with marked regional imbalances in urban green space provision capacity, green space quality, sports facility adequacy, and residents’ access to physical activity. These variations may cause the strength of the association between urban green spaces and healthy aging to diverge across different regions. Classifying areas as high-income or low-income based on provincial per capita disposable income levels provides a more accurate reflection of how economic foundations modulate the health benefits of green spaces.

High-income regions possess robust fiscal capacity and advanced urban planning and construction standards. Their urban green spaces not only demonstrate sufficient quantity but also exhibit significant advantages in functional zoning, accessibility, barrier-free design, and provision of sports facilities. Residents in these areas exhibit higher rates of physical activity alongside more proactive health awareness and lifestyles. Consequently, the comprehensive benefits of green spaces—including environmental quality enhancement, support for outdoor activities, and facilitation of social interaction—are more readily translated into tangible gains for healthy aging. Consequently, green spaces in high-income areas often exert a stronger health-promoting effect. In contrast, low-income areas still face challenges in urban green space development and management, including inadequate infrastructure, insufficient functional completeness, and inefficient use of activity spaces. The utilitarian function of green spaces in these areas is largely confined to basic ecological compensation, struggling to effectively stimulate physical participation among residents, particularly the older adults. Moreover, the scarcity of public sports resources in low-income areas limits residents’ opportunities for physical activity, hindering the full behavioral incentive effect of green spaces. Consequently, health improvements primarily stem from the marginal effects of physical activity itself, rather than the comprehensive benefits of the green environment. Based on this analysis, it can be concluded that the level of economic development influences the extent to which urban green spaces contribute to healthy aging. Accordingly, the following hypothesis is proposed:

*H4*: The association between urban green spaces and healthy aging among older adults exhibits significant variation across regions of differing income levels, with the positive correlation being more pronounced in high-income areas.

## 3 Materials and methods

### 3.1 Data sources

This study utilized individual survey data from the CGSS for the 2010, 2011, 2012, 2013, 2015, 2017, 2018, 2021, and 2023, alongside data from the China Statistical Yearbook (2010–2023), the China Environmental Statistical Yearbook, and municipal statistical yearbooks (e.g., Beijing) to empirically examine the mechanisms through which urban green spaces and physical activity influence healthy aging. The CGSS provides key indicators annually, including residents’ physical exercise behavior, self-rated health, mental health, social participation, and environmental satisfaction. Sample data for individuals aged 60 and above were derived by subtracting the sample’s birth year from the survey year. Based on this, individual-level older adults data were grouped by province and year to calculate provincial averages for relevant variables, constructing panel data at the ‘province-year’ level to examine physical activity levels and healthy aging status among older adults populations across regions. Macro-level data were sourced from multiple statistical yearbooks, encompassing indicators such as urban population density, per capita urban road area, per capita disposable income, green space coverage in built-up areas, health expenditure, environmental protection expenditure, sulfur dioxide emissions, and healthcare facility beds per thousand population. This enables a systematic provincial-level characterization of the relationships between economic development levels, infrastructure provision, environmental governance capacity, and residents’ health behaviors.

In terms of data processing, given the persistent absence of indicators concerning physical activity and physical and mental health across multiple waves in regions such as Tibet, Xinjiang, and Hainan within the CGSS, it proved difficult to establish stable time-series data. Consequently, these regions were excluded from the analysis. Concurrently, the CGSS omitted questions regarding satisfaction with the natural environment in 2011, 2018, and 2023. Furthermore, after filtering samples aged 60 and above, certain provinces lacked valid observations for specific years. This resulted in missing indicators for physical activity and healthy aging in Shanghai (2021), Yunnan (2021), Inner Mongolia (2010, 2011, 2023), Jilin (2021), Tianjin (2021), Ningxia (2010, 2011, 2023), Guangdong (2021), Jiangsu (2023), Guizhou (2021, 2023), and Heilongjiang (2021, 2023). Additionally, statistical gaps exist in sulfur dioxide emissions data for 2018. To address these omissions, linear interpolation was applied according to variable characteristics. Following systematic data cleansing and integration, a provincial panel dataset was ultimately constructed covering 28 provinces and 9 observation periods, comprising 252 valid observations.

### 3.2 Variable descriptions

1) Dependent variable: Healthy aging. Drawing upon research by Chen Hong (35) and Sun Ju (36), a composite index was constructed based on three dimensions from the CGSS: physical health, mental health, and social participation. The sum of these three components reflects the level of healthy aging among the older adults population at the provincial level. During data processing, age was first calculated based on the survey year and the respondent’s year of birth, retaining only samples aged 60 years or older. Sample sizes for older adults respondents varied across provinces and years, typically ranging from over ten to over a hundred in earlier years. After 2018, sample sizes increased to over a hundred, remaining within a reasonable range overall.
2) Explanatory Variable: Urban Green Spaces. The ‘green space ratio in built-up areas’ serves as an indicator of urban greening levels. Drawing upon research by Hong Shunfa et al. (37), the green coverage rate in built-up areas is employed to measure the supply of urban green spaces, reflecting both urban greening quality and ecological infrastructure standards.
3) Mediating Variable: Participation in Physical Activities. Based on the CGSS data, the frequency of residents’ physical exercise is reverse-scored and averaged by province to characterize the level of physical activity among the older adults in each region.
4) Control variables: To minimize the impact of omitted variable bias on estimation results, this study incorporates multidimensional control variables into the baseline model. Drawing upon the research framework of Huang Qian et al. (38) concerning the relationship between physical activity and health wellbeing, and integrating literature on urban environments and public health, controls are applied across multiple dimensions including socioeconomic conditions, infrastructure levels, public health resource provision, and environmental governance. Specifically, these include: urban population density, per capita urban road area, and per capita disposable income to characterize regional economic development and built environment characteristics; regional public health expenditure and healthcare institution beds per thousand population to measure regional healthcare resource investment and health service provision capacity; regional environmental protection expenditure and sulfur dioxide emissions to reflect environmental governance intensity and pollution pressure; additionally, resident satisfaction with the natural environment was incorporated as a subjective environmental perception variable (see Table 1). Through multidimensional control, the study sought to mitigate potential confounding effects of regional economic development, environmental quality, and public service provision on the relationship between urban green spaces and healthy aging.

**Table 1:** Detailed description and calculation methods of relevant variables.

**Variable type**	**Variable name**		**Indicator definition and data source**
Independent variable	Urban green space		The quality of urban greening and the level of ecological infrastructure are measured using the built-up area greening coverage rate (%) indicator published in the China Statistical Yearbook.
Control variable	Urban Population Density		Urban congestion is measured using the urban population density (persons per square kilometer) published in the China Statistical Yearbook.
	Per capita urban road area		The level of urban infrastructure development is measured using the per capita urban road area (square meters) published in the China Statistical Yearbook.
	Per capita disposable income		Residents’ economic level, measured by per capita disposable income (RMB) published in the China Statistical Yearbook.
	Satisfaction with the natural environment		Residents’ subjective perception of natural environment quality is measured using the CGSS item ‘I am satisfied with the natural environment around me’. The original item comprises responses ranging from ‘Strongly disagree’ to ‘Strongly agree’ (coded 1–6), with higher values indicating greater satisfaction with the surrounding natural environment. The sample mean reflects the overall subjective evaluation level of natural environment quality among older adults individuals across provinces.
	Regional public health expenditure		The level of regional public health resource investment is measured using regional public health expenditure (in billions of yuan) as published in the China Statistical Yearbook.
	Regional environmental protection expenditure		The level of investment in regional environmental governance and ecological conservation is measured using environmental protection expenditure (in billions of yuan) as published in the China Statistical Yearbook.
	Sulfur dioxide emissions		The degree of regional environmental pollution is measured using sulfur dioxide emissions (in 10,000 tonnes) as published in the China Statistical Yearbook.
	Number of hospital beds per thousand people		The provision capacity of regional healthcare services is measured using the number of healthcare institution beds per thousand inhabitants (in ten-thousands), as published in the China Statistical Yearbook.
Mediating variable	Physical activity		Physical exercise frequency was measured using the CGSS item: ‘In the past year, how often did you engage in physical exercise during your free time?’ The original response options were “daily,” ‘several times a week’, ‘several times a month’, ‘several times a year or less’, and ‘never’ (original codes 1–5). Frequency was recoded from low to high as scores 1–5 (1 = never, 5 = daily), with higher values indicating greater participation levels. The sample mean was calculated to reflect physical activity engagement among older adults across provinces.
Dependent variable	Healthy aging	Mental health	Residents’ self-rated psychological state was measured using the CGSS item: ‘Over the past four weeks, how frequently have you felt depressed or down?’ The original response options were ‘Always’, ‘Often’, ‘Sometimes’, “Rarely”, ‘Never’ (coded 1–5). Higher values indicate lower frequency of depressive or low mood, signifying better psychological well-being. The sample mean was used to reflect the overall psychological health of older adults across provinces.
		Physical health	Self-assessment of physical health status is measured based on the CGSS item ‘How would you rate your current physical health?’. The original response options are ‘Very poor’, ‘Fairly poor’, “Average,” ‘Fairly good’, and ‘Very good’ (coded 1–5). Higher values indicate better physical health. The sample mean is used to reflect the overall physical health level of older adults across provinces.
		Social participation	Level of participation in social activities, based on the CGSS item ‘In the past year, how often did you meet friends in your free time?’ ‘The original response options were “daily,” ‘several times a week‘, ‘several times a month‘, ‘several times a year or less‘, and ‘never” (coded 1–5). These were recoded from low to high participation frequency as scores 1–5 (1 = never, 5 = daily), with higher values indicating greater social engagement. The sample mean was calculated to reflect the overall level of social participation among older adults in each province.

All the variable attributes mentioned above are continuous variables.

### 3.3 Research methodology

To examine whether urban green spaces influence residents’ healthy aging levels, this study constructs the following benchmark model within a provincial panel data framework:

1) Healthy aging benchmark model

$$\begin{array}{l} \mathrm{ag}e_{i,t}=\beta 0+\beta_{1}\cdot \text{Gree}n_{i,t}+\beta_{2}\cdot \text{Contro}l_{i,t}+\\ \lambda_{i}+\delta_{t}+\mu_{i,t} \end{array}$$ (1)

where i denotes province and t denotes year; age_*i,t*_ represents the healthy aging index, calculated as the sum of standardized scores for mental health, physical health, and social participation; Green_*i,t*_ denotes the urban green space variable; Control_*i,t*_ denotes control variables, Including urban population density, per capita urban road area, etc.; λ_*i*_ represents provincial fixed effects; δ_*t*_ denotes annual fixed effects; μ_*i,t*_ is the error term.

To further examine the mediating role of physical activity between urban green spaces and healthy aging, this study employs a formal test within a two-way fixed-effects model framework. This combines the Baron–Kenny (1986) mediation analysis approach with bootstrap confidence intervals for indirect effects.

First, following the path specifications of the Baron–Kenny method, the following models are sequentially estimated:

2) The effect of urban green spaces on physical activity participation (path a):

$$\begin{array}{l} \text{spor}t_{i,t}=\gamma 0+\gamma_{1}\cdot \text{Gree}n_{i,t}+\gamma_{2}\cdot \text{Contro}l_{i,t}+\\ \lambda_{i}+\delta_{t}+\varepsilon_{i,t} \end{array}$$ (2)

3) Health ageing model incorporating physical activity (path b and direct effect c′):

Where λ_*i*_ and δ_*t*_ denote provincial and annual fixed effects respectively, with standard errors cluster-adjusted at provincial level.


$$\begin{array}{l} \mathrm{ag}e_{i,t}=\theta_{0}+\theta_{1}\cdot \text{Gree}n_{i,t}+\theta_{2}\cdot \text{spor}t_{i,t}+\\ \;\theta_{3}\cdot \text{Contro}l_{i,t}+\lambda_{i}+\partial_{t}+\eta_{i,t} \end{array}$$ (3)

On this basis, indirect effects are calculated via path coefficient multiplication: *Indirect = a × b.*

To circumvent the reliance on normality assumptions inherent in traditional Sobel tests, this study further employs bootstrap sampling (1,000 iterations) to estimate indirect effects, constructing 95% confidence intervals. Mediating effects are deemed significant if the confidence interval excludes zero.

## 4 Results

### 4.1 Baseline regression analysis

The baseline regression results for the impact of urban green space on healthy aging are presented in Table 2. Column 1 presents the baseline regression results without any control variables and without controlling for annual fixed effects. Column 2 adds control variables including population density, per capita road area, per capita disposable income, and satisfaction with the natural environment to Model 1, but still excludes annual fixed effects. Column 3 further incorporates annual fixed effects into Model 2 to eliminate the impact of macro-level annual shocks.

**Table 2:** Benchmark regression results.

Variables	(1)	(2)	(3)
	Healthy Aging	Healthy Aging	Healthy Aging
Urban green space	0.235*** (0.034)	0.130*** (0.031)	0.122*** (0.036)
Urban population density	—	−0.000 (0.000)	−0.000 (0.000)
Road area per capita	—	0.042** (0.016)	0.002 (0.032)
*Per capita* disposable income	—	−0.000 (0.000)	−0.000 (0.000)
Natural environment satisfaction	—	−0.303*** (0.095)	−0.028 (0.175)
Regional public health expenditure	—	0.001** (0.001)	0.001 (0.001)
Regional environmental protection expenditure	—	0.001 (0.001)	0.001 (0.001)
Sulfur dioxide emissions	—	−0.003* (0.001)	−0.003 (0.002)
Number of hospital beds per thousand people	—	−0.008 (0.021)	−0.010 (0.024)
Constant	−1.388 (1.347)	2.738** (1.232)	3.376* (1.730)
Time fixed effects	No	No	Yes
Observations	252	252	252
*R* ^2^	0.349	0.451	0.518

*, **, and *** denote significance levels of 10%, 5%, and 1% respectively; figures in parentheses denote robust standard errors, as below.

Results indicate that across Models 1–3, regardless of whether control variables were included or annual fixed effects controlled for, urban green space exhibited a statistically significant positive association with healthy aging among residents (*β* = 0.235 → 0.130 → 0.122). This association passed the 1% significance level test, confirming a stable positive relationship between urban green space coverage and healthy aging among older adults, thereby validating Hypothesis H1.

Regarding control variables, population density, per capita disposable income, environmental protection expenditure, and number of hospital beds showed no overall significant impact. Per capita urban road area and satisfaction with natural environment demonstrated significance in Model 2 but became unstable after controlling for time fixed effects. Public health expenditure exhibited a significant positive effect in some models, while sulfur dioxide emissions showed a significant negative effect. This indicates that healthcare investment contributes to enhancing healthy aging among the older adults, whereas environmental pollution may exert an inhibitory effect.

### 4.2 Mediating effect test

To further identify the mediating mechanisms through which physical activity links urban green spaces and healthy aging, this study formally decomposes direct and indirect effects within a two-way fixed-effects framework. Path coefficients, standard errors, significance levels, and 95% confidence intervals are reported. Unless otherwise specified, all estimates are based on cluster-robust standard errors at the provincial level. Table 3 presents the total effect of urban green spaces, its direct effect, and the indirect effect mediated through physical activity participation, thereby systematically delineating the pathways through which urban green spaces influence healthy aging.

**Table 3:** Mediation regression results.

**Pathways and effects**	**Coefficient**	**Standard error**	***Z*-value**	***P*-value**	**95% Confidence interval**
Total effect (c)	0.122	0.036	3.38	*P* < 0.01	[0.048,0.196]
Direct effect (c′)	0.102	0.037	2.79	*P* < 0.01	[0.027,0.177]
Pathway a: urban green space → physical activity	0.029	0.011	2.56	*P* < 0.05	[0.006,0.053]
Pathway b: physical activity → Healthy aging	0.695	0.191	3.64	*P* < 0.01	[0.303,1.087]
Indirect Effect (a × b)	0.020	0.009	2.16	*P* < 0.05	[0.002,0.039]

The overall effect estimates reveal that urban green spaces exert a significant positive influence on healthy aging (*β* = 0.122, *p* < 0.01, 95% CI [0.048, 0.196]). This indicates that even after controlling for region-specific fixed effects, year-specific fixed effects, and relevant covariates, urban green spaces remain a crucial factor affecting healthy aging. The mediation path analysis revealed that urban green space significantly promotes participation in physical activity (path *a*: *β* = 0.029, *p* < 0.05, 95% CI [0.006, 0.053]), supporting Hypothesis H2. Furthermore, physical activity participation exhibited a significant positive correlation with healthy aging (path b: *β* = 0.695, *p* < 0.01, 95% CI [0.303, 1.087]), indicating that physical activity participation serves as a crucial behavioural mechanism promoting healthy aging.

Building upon this, the study formally decomposed the effects. Results indicate that the direct effect (c′) of urban green spaces on healthy aging was 0.102 (*p* < 0.01, 95% CI [0.027, 0.177]), remaining statistically significant; while the indirect effect (a × b) was 0.020 (*p* < 0.05, 95% CI [0.002, 0.039]), with the confidence interval not containing zero. This indicates that physical activity participation significantly mediates the relationship between urban green space and healthy aging. Thus, urban green spaces not only promote healthy aging through direct pathways but also exert indirect effects by enhancing physical activity participation, thereby supporting Hypothesis H3.

### 4.3 Heterogeneity analysis

To further examine the influence of economic development levels on the relationship between urban green space and healthy aging, this study divided the sample into high-income and low-income provinces based on the median per capita disposable income of each province, conducting fixed-effects regression analyses for each group. Results indicate that the impact of urban green space on healthy aging exhibits significant differences across income regions.

In high-income areas, urban green spaces exerted a significant positive influence on healthy aging (*β* = 0.178, *p* < 0.01). Conversely, in low-income areas, although the coefficient for urban green spaces was positive (*β* = 0.026), its effect failed to reach statistical significance (*p* > 0.10). In contrast, the promotional effect of physical activity on healthy aging was more pronounced in low-income areas (*β* = 0.750, *p* < 0.05) than in high-income areas (*β* = 0.678, *p* < 0.05). These findings indicate that the statistical association between urban green space and healthy aging exhibits significant regional variation across income levels, with the positive correlation being more pronounced in high-income areas. Hypothesis H4 is thus supported (Table 4).

**Table 4:** Results of heterogeneity analysis.

Variables	High-income regions	Low-income regions
	Healthy aging	Healthy aging
Urban green space	0.178*** (0.027)	0.026 (0.067)
Physical activity	0.678* (0.267)	0.750* (0.303)
Urban population density	−0.000** (0.000)	−0.000 (0.000)
Road area per capita	−0.001 (0.043)	0.012 (0.026)
*Per capita* disposable income	−0.000 (0.000)	0.000 (0.000)
Natural environment satisfaction	−0.151 (0.199)	0.078 (0.255)
Regional public health expenditure	0.002** (0.001)	−0.001 (0.002)
Regional environmental protection expenditure	−0.001 (0.002)	0.002 (0.002)
Sulfur dioxide emissions	−0.005** (0.002)	−0.001 (0.003)
Number of hospital beds per thousand people	−0.024 (0.046)	0.026 (0.035)
Constant	0.821 (1.753)	2.377 (3.948)
Province and time fixed effects	Yes	Yes
Observations	126	126
*R* ^2^	0.614	0.622

### 4.4 Robustness tests

To validate the robustness of the benchmark regression results, this study conducted robustness tests across three dimensions: replacing the core explanatory variable, replacing the core dependent variable, and altering the model specification (see Table 5). Firstly, regarding the replacement of the core explanatory variable, following established research practices, the regression was re-run using per capita green space area in cities as a substitute for green space coverage in urban built-up areas. Results indicate that the estimated coefficient for per capita green space remains significantly positive at the 5% statistical level, with a direction consistent with the benchmark model. This demonstrates that differing metrics for green space provision do not materially alter the study’s conclusions. Secondly, regarding the replacement of the core dependent variable, considering that healthy aging indicators may be influenced by scaling effects and distribution skewness, this study substituted it with both the logarithmic form ln(age) and the standardized indicator age_z, re-estimating using the same model. Results indicate that the estimated coefficients for both substitute indicators remain significantly positive at the 5% statistical level, further validating that altering the construction method of healthy aging indicators does not affect the direction or significance of the green space effect. Thirdly, to test the robustness of the model specification, a random effects (RE) model was employed for comparative analysis. Regression results indicate that the estimated coefficient for the urban green space variable remains significantly positive at the 1% level. Moreover, both the significance level and coefficient magnitude are highly consistent with those from the two-way fixed effects model, suggesting that changes in model specification do not alter the primary conclusions. Finally, to examine the impact of the healthy aging index construction method on results, principal component analysis (PCA) was employed to statistically integrate the three dimensions of physical health, mental health, and social participation. Results indicated that the first principal component explained 59.92% of the total variance, effectively reflecting the comprehensive characteristics of healthy aging. Following the construction of an alternative indicator based on this principal component, a two-way fixed effects regression was conducted. The estimated coefficient for urban green space was 0.121 (*p* < 0.01), maintaining both directionality and statistical significance consistent with the benchmark model. This demonstrates robust stability of the research conclusions across different index construction methods. In summary, whether replacing core explanatory variables, core dependent variables, or adjusting model specifications and index construction methods, the directional effect of urban green space on promoting healthy aging remained consistent and significant. This demonstrates the robust validity of the benchmark regression results.

**Table 5:** Robustness test results.

Variables	(7) Replace explanatory variable p_green	(8) ln(age)	(9) age_z	(10) Random effect RE	(11) Replace method for constructing healthy aging index
Urban green space	0.208^***^ (0.038)	0.016^**^ (0.005)	0.107^**^ (0.032)	0.079^***^	0.121***
Control variables	NO	Yes	Yes	Yes	Yes
Intercept	5.198^***^ (0.505)	1.489*** (0.235)	−3.991** (1.515)	4.877*** (1.253)	−4.361*** (1.520)
Time/region fixed effects	NO	Yes	Yes	Yes	Yes
Observations	252	252	252	252	252
*R* ^2^	0.230	0.529	0.538	0.529	0.567

## 5 Discussion

### 5.1 Key findings and interpretation

Firstly, concerning RQ1, the study found a significant positive correlation between urban green space levels and healthy aging among older adults. This result supports the fundamental tenets of environmental behavioral science and ecological health theory regarding the long-term health-promoting effects of natural environments, aligning with existing green space health research. Previous studies indicate that urban green spaces exert positive impacts on residents’ physical and mental health through ecological regulation functions such as improving microclimate conditions, reducing air pollution levels, and mitigating the urban heat island effect (39). Concurrently, Xu T (2024) (40), based on a sample of Nanjing residents aged 60 and above, found that the spatial structure and functional characteristics of urban green spaces significantly enhance older adults’ subjective well-being. Moreover, the influence of certain spatial quality factors even surpasses that of purely natural attribute indicators. Urban green spaces not only improve individual health status in the short term but may also support the maintenance of functional capacity among older adults over longer time scales. Mechanistically, the health benefits of urban green spaces may operate through multiple pathways. Firstly, ecological regulation functions mitigate risks to the cardiovascular and respiratory systems posed by heat exposure and air pollution among the older adults (41), thereby strengthening foundational physical health. For instance, vegetation within urban green spaces absorbs harmful gases and releases oxygen, effectively enhancing air quality – a factor particularly crucial for supporting respiratory and cardiovascular health in older adults. Secondly, as open and accessible public spaces, green areas provide venues for daily activities and social interaction among older adults, alleviating loneliness and psychological stress while enhancing emotional resilience. Compared to studies emphasizing the role of ‘green space coverage,’ this research demonstrates that urban green spaces transcend their function as ecological resources. They constitute vital infrastructure for promoting the physical and mental wellbeing of older adults and enhancing their quality of life.

Secondly, concerning RQ2, the study found a significant positive correlation between urban green space levels and physical activity participation among older adults. This aligns with behavioral ecology theory, which posits that environmental factors—particularly green space accessibility, spatial layout, and activity facilities—significantly influence residents’ engagement in physical activities (42). Existing research, predominantly based on community-level or individual survey data, emphasizes the role of green space accessibility, greenway development, and facility provision in promoting physical activity frequency. For instance, Deng et al. (2023) noted in their systematic review and meta-analysis that urban greenway construction significantly enhances residents’ physical activity levels, underscoring the necessity of greenways as effective public health interventions (43). From a mechanistic perspective, urban green spaces create supportive environments for older adults physical participation by reducing environmental costs associated with exercise. Firstly, green spaces provide open, low-threshold exercise areas. Against the backdrop of declining physical capacity and safety concerns among older adults, this effectively lowers participation barriers and enhances behavioral willingness. Secondly, infrastructure such as pathway networks, sports facilities, and shaded amenities enhance activity safety and comfort, thereby improving the sustainability of physical engagement. Thirdly, good spatial accessibility reduces reliance on transport, enabling older adults to undertake regular outdoor activities within their daily living radius, thus increasing exercise frequency and behavioral consistency.

Furthermore, concerning RQ3, the study found that physical participation partially mediates the relationship between urban green spaces and healthy aging. This outcome reveals the intrinsic logic of ‘environment, behavior, health’ at the behavioral pathway level, strongly resonating with existing literature. Hansen Li et al. (44) constructed an analytical framework of ‘green spaces, green physical activities, mental health’ based on an urban resident sample, indicating that recreational green physical activities significantly mediate the relationship between green spaces and mental health, whereas commuting physical activities exerted no significant effect. From a mechanistic perspective, physical activity constitutes a vital behavioral bridge converting urban green spaces into health benefits. On one hand, regular physical activity significantly improves older adults’ cardiopulmonary function, maintains muscle strength and bodily coordination, reduces chronic disease and fall risks, thereby delaying functional decline and enhancing healthy aging outcomes (45). On the other hand, physical activity possesses significant psychological adaptation and social integration functions. Older adults individuals engaging in group activities such as Tai Chi, square dancing, or brisk walking within green spaces not only enhance physical fitness but also strengthen social connections through collective interaction. This alleviates loneliness and psychological stress, elevating life satisfaction and subjective well-being. Particularly within an ageing context, social support and emotional resilience have been confirmed as crucial dimensions influencing healthy aging. It is worth emphasizing that this study found physical participation to exert only a partial mediating effect. This implies that the impact of urban green spaces on healthy aging does not rely solely on the physical activity pathway. Instead, it may exert direct effects through multiple ecological and psychological mechanisms, including improved air quality, microclimate regulation, enhanced environmental security, and strengthened emotional recovery.

Finally, concerning RQ4, the study also found that the impact of urban green spaces on healthy aging varies across different income levels, exhibiting distinct socio-economic stratification characteristics. This outcome aligns closely with the social stratification perspective within the ecological health model. The underlying reason is that in high-income areas, local governments invest more substantially in optimizing green space functionality, facility maintenance, safety management, and accessible design. Consequently, green spaces not only serve aesthetic and recreational purposes but also effectively facilitate physical activities and social interaction, thereby more readily transforming into comprehensive health resources that enhance older adults’ physical function, mental wellbeing, and social engagement. Concurrently, older adults in high-income areas possess advantages in health literacy and exercise awareness, making it easier for them to establish regular physical activity habits, thereby amplifying the health-promoting effects of green spaces. In contrast, while low-income areas have seen an increase in green space quantity, factors such as substandard facilities, inadequate maintenance, and poor accessibility constrain the fulfilment of these spaces’ potential, hindering the full realization of health benefits. Existing research indicates that the functional development of green spaces is a stronger determinant of health benefits than mere surface area, and such functional optimization often relies on stable public investment (46). Consequently, quantity-driven expansion alone cannot effectively improve health outcomes in low-income areas. Greater emphasis should be placed on facility enhancement and community support system development to narrow regional health disparities. From the perspective of physical activity participation mechanisms, the positive correlation between physical activity engagement and healthy aging is more pronounced in low-income areas, demonstrating a compensatory effect of increasing marginal returns. Given their lower baseline activity levels, modest increases in physical activity yield substantial health improvements. Conversely, in high-income areas where participation levels are already relatively high, marginal health gains tend to diminish. Furthermore, in high-income regions, population density and sulfur dioxide emissions exert significant negative impacts, while regional public health expenditure exerts a significant positive influence. This indicates that in economically advanced regions, environmental carrying capacity and public health resource investment are equally crucial structural factors influencing healthy aging. On the one hand, high-density urban environments may diminish the health benefits of green spaces and physical activity through spatial crowding and environmental stress. On the other hand, stable public health investment strengthens health support systems, while pollution emissions may offset some of the health gains derived from green spaces. This highlights the significant moderating role of environmental governance and health investment in high-income regions.

### 5.2 Limitations

Although this study systematically analyses the relationship between urban green spaces, physical activity, and healthy aging using provincial-level panel data from 2010 to 2023, revealing structural associations to some extent, several limitations remain that warrant further exploration in future research.

First, regarding causal identification, while this study minimized the risk of omitted variables through extended control variables and robustness tests, it remains challenging to entirely exclude the influence of unobserved confounding factors that evolve over time (such as expansions in healthcare resources, or urban renewal policies). These factors may simultaneously affect both urban green space provision and the healthy aging levels of older adults. Consequently, the findings should be interpreted as statistical associations rather than strict causal effects. Secondly, in identifying mediating mechanisms, although path decomposition and bootstrap methods were employed to test indirect effects, the analysis relies on the identification assumption that ‘no unobserved confounders exist between mediators and outcomes.’ Furthermore, primary variables were measured in the same time period, limiting temporal sequence identification capabilities. Consequently, the mediating role of physical activity should be understood more as a structural association pathway rather than a strict causal mediating effect. Finally, certain limitations persist in variable measurement. Urban green space was measured using built-up area greening coverage, a macro-level proportion indicator that struggles to capture spatial distribution patterns, accessibility, and functional quality variations. Moreover, the healthy aging index, constructed from self-reported physical health, depression frequency, and social participation, primarily reflects subjective overall health status without incorporating objective clinical indicators such as cardiovascular function or cognitive abilities. Consequently, the study’s findings should not be interpreted as direct evidence of improvements in specific physiological or neurocognitive functions.

### 5.3 Policy implications

Urban green spaces serve as both vital vehicles for ecological civilization development and key public health resources for promoting physical participation and healthy aging. To further harness the value of urban green spaces in fostering healthy aging, the following recommendations are proposed across spatial development, facility provision, regional equity, and behavioral cultivation:

#### 5.3.1 Optimize the spatial layout of urban green spaces to enhance accessible and user-friendly green ecological environments

Research indicates that the contribution of urban green spaces to healthy aging hinges on whether older adults can conveniently and consistently access and utilize these spaces. Consequently, urban renewal and spatial planning should systematically optimize the spatial layout of green spaces, centering on older adults’ daily mobility capabilities and activity patterns.

Firstly, prioritize walkability by enhancing the provision of community-scale green spaces. In urban planning practice, incorporate the daily walking capacity of older residents as a key constraint in green space allocation. Give priority to establishing pocket parks, micro-green spaces and neighborhood parks around residential areas, thereby fostering the development of a 15-min fitness ecosystem accessible to all. For instance, Jinjiang District in Chengdu implemented a small-scale green space expansion project within high-density residential areas. By utilizing vacant street-side plots, idle corner lots, and spaces outside public institution walls, 63 pocket parks and micro-green spaces were created. This significantly increased the proportion of residents who can ‘see greenery upon stepping out and reach green spaces within five minutes’. Secondly, integrate fragmented spaces to construct a continuous, accessible green activity network. Within high-density built-up areas and older communities, supplement internal green space provision by revitalizing street-side green areas, marginal plots, building rooftops, elevated structures, and unused public spaces. Connect these dispersed green spaces organically through greenways, slow-traffic systems, and secure pedestrian corridors, forming a continuous, accessible, and circular green activity network. Thirdly, during accessibility optimization, concurrently prioritize safety, low maintenance requirements, and comfort to enhance the actual utilization rate of green spaces. In site selection and spatial organization, traffic interference should be reasonably controlled. Accessibility facilities and safety lighting systems should be improved to reduce mobility risks for older adults. Local microclimates can be enhanced through plant selection, terrain shaping, and water feature creation, mitigating adverse effects of thermal stress, noise, and air pollution on older adults sports activities. Simultaneously, comfort should be elevated through shaded areas, seating, and gentle slopes. Integrating ecological restoration further boosts comfort, safety, and health benefits within green spaces.

#### 5.3.2 Promoting the sports-oriented transformation of urban green spaces to establish integrated shared fitness environments

When urban green spaces accommodate sporting activities, their ecological health benefits can translate into tangible health gains for older adults, thereby enhancing healthy aging outcomes. Consequently, efforts should be made to advance the sports-oriented transformation of urban parks, incorporating the promotion of physical activity as a key objective in optimizing green space functionality. This will foster a public space system where green areas, physical activity, and health form a synergistic and interconnected whole.

Firstly, institutional embedding should drive the routine implementation of sports-oriented renovations in urban park green spaces. Integrate such renovations into urban renewal, old residential area refurbishment, and local national fitness programs. Clarify the responsibilities of departments such as urban construction and sports in planning, construction, and management, while establishing construction standards and operational norms. For instance, Wuhan’s East Lake Scenic Area has integrated ‘greenways + parks + sports venues’ to create approximately 105 kilometers of lakeside greenways. These connect multiple lakeside parks and host events like marathons and cycling, transforming scenic green spaces into sports and wellness areas that significantly boost residents’ daily participation in physical activities. Secondly, enhance the sports capacity of urban park green spaces with a focus on promoting participation. At the planning and design level, the traditional approach prioritizing ornamental features in park construction should be abandoned. The proportion of space and paved areas suitable for physical activity within park green spaces should be appropriately increased. Fitness trails, slow-moving systems, simple strength training facilities, and older adults-friendly fitness equipment should be scientifically arranged to support diverse, low-to-moderate intensity physical activities for older adults of varying fitness levels. Simultaneously, through functional zoning and spatial guidance, the relationship between static rest and dynamic exercise should be reasonably balanced, driving the transformation of urban parks from being ‘landscape-centric’ to ‘usage-centric’. Thirdly, enhance the sports service efficacy of small and medium-sized park green spaces through differentiated provision. For compact urban parks, tailor configurations to surrounding demographics and older adults activity needs—such as prioritizing walking circuits, flexibility training zones, or rehabilitative exercise facilities. Connect multiple park green spaces via greenways, slow-traffic systems, and footpaths to form a multi-point, functionally complementary sports activity network.

#### 5.3.3 Coordinating the balanced provision of green health resources to strengthen the cultivation of healthy aging behaviors among the older adults

Research indicates that the health-promoting effects of increased urban green spaces on healthy aging depend not only on the level of spatial and facility provision but also on the actual usage behavior of the older adults population. Therefore, the provision of green health resources should be advanced in tandem with the cultivation of residents’ behavioral habits to amplify their health-promoting effects on ageing.

Firstly, promote the balanced provision of urban green space resources across regions. Addressing existing disparities in urban green space and sports facility provision across China’s diverse regions, public finance allocation, urban–rural development, and territorial spatial planning should establish a resource allocation orientation favoring underserved areas. This will foster a green health space network that is comprehensive, well-connected, and equitably accessible, ensuring provision of green spaces and sports facility land in densely populated areas, low-income communities, older urban districts, and urban–rural fringe zones. For instance, Baiyun District in Guangzhou addressed deficiencies in public spaces within older neighborhoods by constructing greenways, community sports parks, and public fitness centers at urban–rural interfaces. This provided nearby residents with more accessible environments for walking, exercise, and leisure, thereby promoting balanced green resource provision in the area. Secondly, through multi-stakeholder collaboration, guide the effective conversion of green spaces into venues for healthy aging behaviors. Utilize community health education, older adults sports guidance, grassroots social organization mobilization, and media science communication to promote safe, sustainable outdoor activities such as walking and power walking, thereby cultivating regular exercise habits among older adults. Thirdly, strengthen exemplary leadership and institutional coordination to enhance the sustained utilization of green health facilities. Leveraging policy platforms such as ‘Healthy Cities’, ‘Park Cities’, and the ‘15-min community living circle’, we coordinate the development of green spaces, sports facilities, and ageing health services. Through regular exercise programs and volunteer services, we guide older adults toward consistent use of public fitness spaces, increasing both the frequency of green space utilization and its health-promoting effects, thereby narrowing the gap in healthy aging.

## 6 Conclusion

This study systematically examines the relationship between urban green space, participation in physical activities, and healthy aging among older adults, based on multi-period provincial panel data from 2010 to 2023. Key findings are as follows: (1) Urban green space levels exhibit a significant positive correlation with residents’ healthy aging. This effect remains stable after controlling for multiple factors including population density, road area, income levels, and environmental satisfaction. It persists across various substitutions of urban green space indicators, constructions of healthy aging, and multiple robustness tests. (2) Urban green space shows a significant positive correlation with residents’ physical activity levels. (3) Physical activity participation exhibits a partial mediating pathway between urban green space and healthy aging, indicating that the statistical relationship between green space and healthy aging is partially mediated through the behavioral variable of physical activity. (4) Heterogeneity analysis reveals that in high-income provinces, both urban green space and physical activity are significantly correlated with healthy aging, presenting a structural feature where green space and physical activity jointly influence healthy aging. Conversely, in low-income provinces, the statistical association between urban green space and healthy aging is insignificant, with healthy aging levels more strongly linked to physical activity, exhibiting a predominantly sports-participation-driven association pattern.

## Data Availability

Publicly available datasets were analyzed in this study. This data can be found here: http://cgss.ruc.edu.cn/.
